# Tattooing Plastics with Reversible and Irreversible Encryption

**DOI:** 10.1002/advs.201903785

**Published:** 2020-04-22

**Authors:** Tiwa Yimyai, Treethip Phakkeeree, Daniel Crespy

**Affiliations:** ^1^ Department of Chemical and Bimolecular Engineering School of Energy Science and Engineering Vidyasirimedhi Institute of Science and Technology (VISTEC) Rayong 21210 Thailand; ^2^ Department of Materials Science and Engineering School of Molecular Science and Engineering Vidyasirimedhi Institute of Science and Technology (VISTEC) Rayong 21210 Thailand

**Keywords:** data storage, disulfide bonds, elastomers, information encoding, self‐healing materials

## Abstract

Self‐healing materials are explored for restoring mechanical, electrical, and chemical properties. Inspired by the process of tattooing on human skin, a method for engraving non‐permanent or permanent messages on plastics is developed. A self‐healing polymer containing dynamic disulfide bonds is employed as substrate for encryption of written messages. The polymer is engraved with a dye solution which is subsequently covered by the polymer matrix upon activation with temperature increase. The dye is then located at the subsurface of the substrate so that the information cannot be removed easily by wear or extraction with solvents. Therefore, self‐healing polymers can be applied as sustainable substrates for reversibly and irreversibly engraving information.

## Graphical abstract

Inspired by the process of tattooing on human skin, an intrinsic self‐healing polymer containing dynamic covalent bonds is proposed as a substrate for information storage. In this work, reversible and irreversible data encryption are demonstrated by mechanically writing a message in polymer substrates without and with dyes.

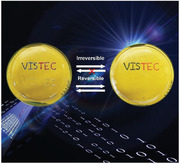

Data storage is nowadays an extremely critical issue due to overwhelming electronic information from digital technologies. The need for data storage capability was 33 zettabytes (33⋅10^21^ bytes) in 2018 and is expected to reach 175 zettabytes in 2025.^[^ ^1^ ^]^ Electronic data storage is associated with consumption of large amount of energy for cooling microprocessors and an increasing demand for space. Miniaturization of storage devices using DNA and synthetic polymers is currently explored to store information in less space.^[^ ^2^, ^3^ ^]^ Sequence‐controlled polymers and DNA can be used for encoding and preserving information because their molecular structure is organized precisely and can be chemically or physically^[^ ^4^, ^5^ ^]^ read. Another challenge is to find sustainable approaches for storing reversibly or irreversibly information on demand. Reversible storage is environmentally friendly because the encoded substrate can be recycled, hence saving energy and resources. Irreversible storage may become sustainable when the lifetime of the encoded substrate is significantly increased so that no new substrates are needed.

This paradox related to sustainability versus lifetime is typically encountered in self‐healing materials,^[^ ^6^, ^7^, ^8^, ^9^, ^10^, ^11^, ^12^ ^]^ that is, materials that can repair themselves. Indeed, the preparation of a highly mechanically durable plastic can be as sustainable as a less robust but self‐healable plastic filling the same purpose. To date, self‐healing materials have been implemented for various applications such as anticorrosion,^[^ ^13^, ^14^ ^]^ antifouling,^[^ ^15^, ^16^, ^17^ ^]^ smart electronics,^[^ ^18^, ^19^, ^20^ ^]^ and energy storage.^[^ ^21^ ^]^ Their self‐healing properties were employed to recover mechanical, chemical, or electronic properties.

Herein, we propose to apply self‐healing materials for reversible and irreversible encryption of information, and more specifically for writing and erasing messages (**Figure** **1**). An intrinsic self‐healing elastomer with dynamic disulfide bonds was used as substrate to store information. The healing mechanism of this self‐healing polymer relies on dynamic disulfide bond exchange reactions^[^ ^22^, ^23^, ^24^, ^25^, ^26^, ^27^ ^]^ that are activated by increase of temperature.^[^ ^27^, ^28^ ^]^ Messages can be mechanically engraved and erased by activating healing of the elastomer. Besides, messages can be also irreversibly encrypted by introducing a dye during engraving of the elastomer. During healing, the dye is sequestrated at the subsurface of the elastomer because the surface energy of the dye is larger than the surface energy of the polymer. Thus, the mechanism of irreversible encoding of information is similar to process of carving tattoos, for which the skin is damaged in the presence of a pigment, followed by healing of the skin and entrapment of the pigment.

**Figure 1:**
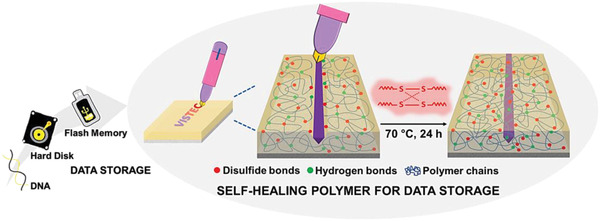
Conceptual scheme for data storage in self‐healing elastomers containing disulfide bonds.

The sentence “This message will self‐destruct in five seconds” is very probably familiar to most of the readers, as well as electrically erasable data storage in memory devices such as memory card, flash memory, and hard disk drives. Herein, erasing data storage was activated by temperature increase. In this work, a self‐healing polymer, that is, a polyurethane containing disulfide bonds (PUDS), was synthesized by polyaddition of polytetrahydrofuran (PTHF) and dicyclohexylmethane 4,4′‐diisocyanate (HMDI) with 2‐hydroxyethyl disulfide (HEDS) as chain extender. The polymer displayed an intrinsic self‐healing ability due to the disulfide bonds in its molecular structure.^[^ ^24^ ^]^ Healing of damaged surface took place by increasing the temperature above glass transition temperature, resulting in enhanced mobility and rearrangement of polymer chains, and enabling dynamic disulfide exchange reaction. For reversible data storage, the self‐healing polymer film was casted on glass petri dishes and words were engraved at 25 °C as shown in **Figure** **2**a. The message was erased by increasing temperature to 70 °C. Different messages, such as week days, could be sequentially engraved, erased, and read on the same elastomeric substrate (Figure S1, Supporting Information). Reversible data storage is achieved by the self‐healing ability of the polymer, which relies on thermal motion of molecular chains and disulfide bond exchange reaction^[^ ^24^, ^25^, ^27^ ^]^ that are promoted upon temperature increase.^[^ ^27^, ^28^ ^]^

**Figure 2:**
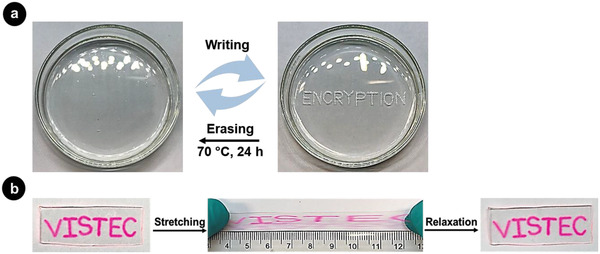
a) Photograph of the self‐healing polymer films after engraving (right) and erasing (left). b) Photograph of healed film engraved with Rhodamine B that was stretched three times with 200% elongation at 25 °C.

We investigated the stability of reversible inscriptions by monitoring the word “STABLE,” which was engraved on a self‐healing polymer film at 23−24 °C and a humidity of 36 ± 4%. The message on the film lasted over at least 24 days (Figure S2a, Supporting Information). Indeed, the dynamic bonds responsible for the self‐healing ability of the film are not mobile enough below the *T*_g_ (glass transition temperature) of the polymer. Besides, self‐healability of engraved polymer film was investigated after an exposure for 24 days. The film was cut, connected, and consequently healed at 70 °C for 24 h. The film was healed and an engraved message disappeared after heat treatment. The polymer film maintained its self‐healing ability after exposure at 23−24 °C and a humidity of 36 ± 4% for 24 days (Figure S2b, Supporting Information). In general, the healing efficiency of materials after several damage/healing cycles can be gradually deteriorated due to imperfect alignment of damaged materials and/or heterogeneous recombination of dynamic bonds of fractured surfaces.^[^ ^29^ ^]^

It should be noted that engraved domains can be healed faster than in 24 h. Defects (28 µm × 4 mm × 2.3 µm) were produced on polymer films using a cutter blade. The engraved polymer films were then healed at 70, 100, and at 70 °C in the presence of solvent (**Figure** **3**a). The healing of the damage was then observed by Raman microscopy and with a profilometer. At 70 °C, the extent of damage in the film gradually decreased until no sign of surface damage was observed after 30 min healing (Figure 3b). Depositing small amount of THF on the engraved surface or healing at 100 °C dramatically reduced the healing time to 5 and 15 min, respectively. Therefore, 24 h are not necessary for a complete healing and the healing can be accelerated by either heating further or adding some solvent to the damaged film. Furthermore, we studied the relationship between self‐healing duration and extent of damage by monitoring the healing process of defects with different sizes. The defects were then monitored in time with a dispersive Raman microscope and a profilometer. The results showed that the large defect healed more slowly than the small defect (Figure S3a, Supporting Information). Indeed, the depth of the large defect decreased to 20% of the initial depth after scratching within 300 min whereas the small defect was fully healed within 30 min (Figure S3b, Supporting Information).

**Figure 3:**
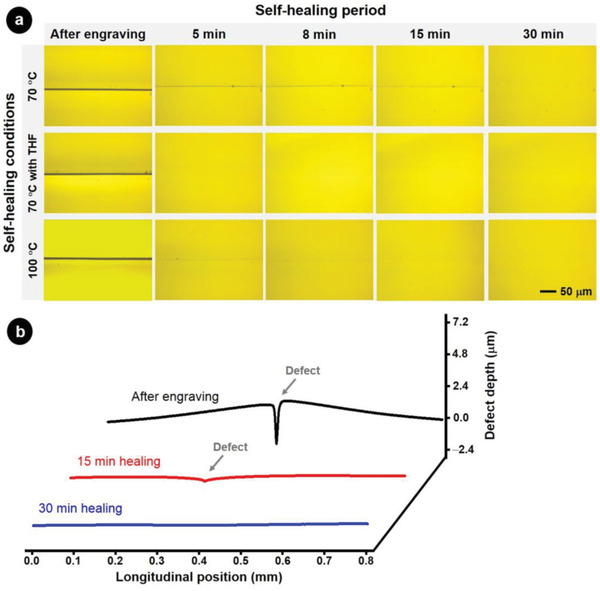
a) Time‐lapse Raman microscopy images of engraved polymer films healed at 70 °C, at 70 °C in the presence of THF, and at 100 °C. b) Temporal evolution of the depth along the length of the defect in the films healed at 70 °C.

The engraving on polymer films was performed using a cutter blade. Furthermore, operating temperatures at the surface of the polymer film during the engraving process should not be above decomposition temperature at  ≈250 °C,^[^ ^24^ ^]^ so that the polymer does not degrade, resulting in maintaining healability. Thus, engraving techniques associated with heat, for example, a laser engraver, are not suitable. Indeed, the temperature at the focal point of a laser beam in a commercial laser for engraving can be above 300 °C.^[^ ^30^ ^]^

In order to automate the engraving processing, a computer numerical control (CNC) drawing machine (AxiDraw V3, Evil Mad Science Laboratories, USA) connected to a tattoo pen was employed (**Figure** **4**a). The CNC machine was controlled with Axidraw extension with the Inkscape software to engrave a specific design, that is, a QR code for the website of our laboratory www.crespylab.com (Figure 4b; Video S1, Supporting Information). A tattoo needle with a diameter of ≈0.3 mm (Figure S11a, Supporting Information) was used for engraving, resulting in an engraving resolution of at least 85 dots per inch (dpi).

**Figure 4:**
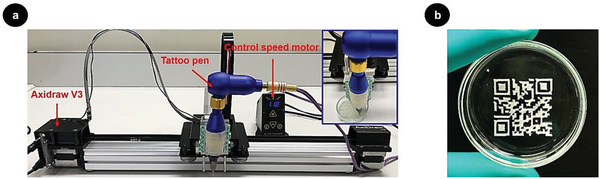
a) Photograph of a computer numerical control (CNC) drawing machine (AxiDraw V3) connected to a tattoo pen, and automatic engraving on a polymer film (inset). b) Photograph of QR code linked with the homepage www.crespylab.com generated by automatic engraving of a CNC drawing machine (AxiDraw V3).

Additionally, we prepared polyurethane containing disulfide bond (PUDS) and polycarbonate‐based polyurethane (PCDS) films with different glass transition temperatures, which were arranged and bonded together (Figure S4a, Supporting Information). The assembled film was used as data storage medium in which two messages could be created and erased by varying temperature.

Thermal properties of PCDS and PUDS were measured by differential scanning calorimetry (DSC) and dynamic mechanical analysis (DMA). As shown in Figure S5, Supporting Information, PCDS displayed a glass transition temperature (*T*_g_) for the polycarbonate soft segment at −39 °C, and two endothermic peaks at 46 and 81 °C corresponding to the melting of crystalline phase in polycarbonate and hard segment, respectively. DMA measurements evidenced a *T*_g_ for the soft segment in polycarbonate displayed at −29 °C and a melting of the crystalline phase in polycarbonate above 39 °C (Figure S6, Supporting Information). The crystalline phase in PCDS was attributed to dipole interaction between carbonate groups on adjacent chains as well as ordering of linear and aliphatic chains in the hard segment.^[^ ^31^, ^32^ ^]^ For PUDS, the transition temperatures were evaluated by combination of DSC and DMA. The *T*_g_ of soft segment in PUDS was displayed at around −50 to −40 °C while the *T*_g_ of hard segment was observed at 25 °C from Figures S5 and S6, Supporting Information.

A polymer film containing PCDS and PUDS was fabricated by joining square pieces of the different polymers (Figure S4a, Supporting Information). A word, “SCIENCE”, was engraved on the assembled film by a cutter blade. The letters were subsequently erased at different temperatures (Figure S4b, Supporting Information). Due to lower *T*_g_ of hard segment in PUDS compared with PCDS, the letters on PUDS region were first erased at 60 °C, leading to a new word, “SINE”. Afterward, the remaining letters could be completely erased at 90 °C. Therefore, the engraved letters could be sequentially erased to yield various messages at different temperatures.

For an irreversible encryption of message on the substrates, we introduced a substance (dye) directly after engraving the substrate. This process is therefore very similar to tattooing, which relies on the mechanical subcutaneous introduction of dye followed by healing of the skin. A solution of dye (0.025 mg mL^−1^), that is, Rhodamine B or Nile red, was deposited into freshly engraved samples and the films were subsequently heated at 70 °C. After self‐healing of the films, the dyes were embedded below the surface of the films. The robustness of irreversible encryption was further tested by stretching substrates. Healed films containing Rhodamine B with a length of 1 cm were stretched three times to 9 cm (200% elongation). The message remained after stretching (Figure 2b), even after different manipulations such as flexing or twisting (Figure S7, Supporting Information).

Conversely to traditional carving or painting, the dye was not anymore at the surface of the substrates but inside them. To confirm this observation, non‐healed and healed films with engraved Rhodamine B dye were immersed for 2 h in water. The appearance of the engraved message did not change for healed samples whereas color was fading in non‐healed samples due to leaching of the dye in water (**Figure** **5**a). A similar phenomenon was observed when the more hydrophobic Nile red was engraved in the film, followed by immersion in *n*‐hexane (Figure S8, Supporting Information). Diffusion of the dye from non‐healed substrates was evident as shown in time‐lapse images (Figure S9, Supporting Information).

**Figure 5:**
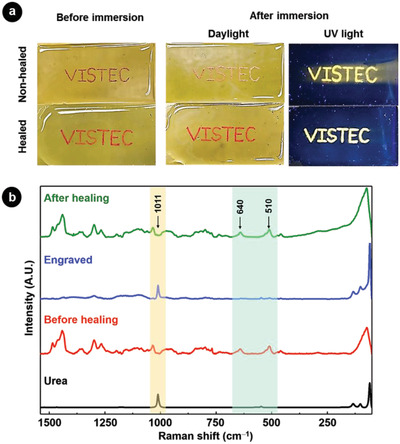
a) Photographs of films engraved with Rhodamine B before and after immersion in water for 2 h. b) Raman spectra of pristine self‐healing substrate (red), the substrate after engraving with urea and before healing (blue), and the engraved substrate after healing (green). The spectrum for pure urea (black) is shown as reference. The characteristic bands of urea (1011 cm^−1^) and the self‐healing polymer (640 and 510 cm^−1^) are highlighted.

In order to investigate the healing of the substrate, we engraved the polymer substrate with urea, which shows a very characteristic band at 1011 cm^−1^ related to N‐C‐N stretching in Raman spectroscopy (Figure 5b). This band was also clearly observed in the region where the substrate is engraved with urea. However, the band vanished after healing the substrate at 70 °C for 24 h. Indeed, only bands for the self‐healing polymer such as S‐S stretching and C‐S stretching at at 510 and 640 cm^−1^ were observed. This indicates that the engraved urea was indeed located beneath the surface of the polymer substrate after healing.

To further verify the irreversible character of the encryption after healing, friction tests on the surface of engraved and subsequently healed films were carried out. For comparison, a non‐healed film obtained from depositing Rhodamine B solution on the surface of a polymer film, and drying at 25 °C for 2 h was used as a control sample. Remarkably, the message in healed films could be still clearly read after 900 polishing cycles (**Figure** **6**a). On the contrary, the message on the non‐healed film faded away (Figure 6b). Under UV light, traces of the dye on the non‐healed surface were observed, which corroborate the observation of vanishing of the dye due to friction.

**Figure 6:**
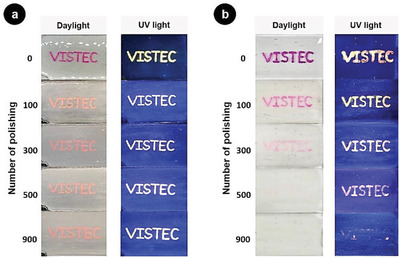
Photographs of a) a healed film and b) a non‐healed film with Rhodamine B after polishing with abrasive paper.

The mechanical properties of engraved polymer substrates before and after healing were investigated by tensile testing. Specimens were prepared as a rectangle shape with a thickness of 0.13 ± 0.03 mm. Polymer films were engraved with a cutter blade, then Rhodamine B solution was deposited in the engraved section of the specimen. The films were subsequently healed at 70 °C in an oven for 24 h before measurement with a strain rate of 100 mm min^−1^ at 24 °C and a humidity of 55 ± 4% (Figure S10, Supporting Information). Pristine films and engraved films without dye were also prepared as control samples. As shown in **Table** **1**, ultimate tensile strength, ultimate tensile strain, and healing efficiency of engraved polymer films slightly decreased compared with pristine films. However, ultimate tensile stress and ultimate tensile strain of the engraved films with dye were comparable with the engraved films without dye. Therefore, the presence of the dye inside the films did not affect significantly the healing ability of the polymer.

**Table 1:** Mechanical properties and healing efficiency of pristine films, engraved films with dye, and engraved films without dye

Entry	Average ultimate tensile strength [MPa]	Average ultimate tensile strain [%]	Healing efficiency [%]
Pristine films	26.9 ± 2.7	629 ± 28	—
Engraved films with dye	23.0 ± 4.7	617 ± 31	85.5
Engraved films without dyes	23.4 ± 1.0	613 ± 52	87.0

The healing efficiency (%) is the ratio of ultimate tensile strength of engraved films to pristine films.

In order to generalize the concept of “plastics tattooing,” we applied our method with a real tattoo pen as shown in Figure S11, Supporting Information. Interestingly, we fabricated a composite coating. The composite coating contained an ink reservoir in which the tattoo ink was filled, a layer of opaque plastic, and a layer of self‐healing polymer as depicted in Figure S12a, Supporting Information. We engraved a message on the polymer film using a tattoo needle. The composite coating was then healed at 70 °C for 15 h to give an irreversible encryption in the film (Figure S12b, Supporting Information). With this technique, an irreversible encryption was successfully prepared without applying ink via a separate step.

Self‐healing of the films occurs via dynamic disulfide bonds exchange. The disulfide bonds are present in the hard phase of the polyurethane so that healing can be activated upon temperature increase above the glass transition temperature. Because microphase separation between the domains occurs at the nanoscale, the elastomeric films are transparent. When dyes are engraved in the films, the polymer is covering the dyes because it has a lower surface energy than the dyes. Surface free energy of self‐healing polymer were estimated to be 25.5 mJ m^−2^ with the Owens/Wendt theory (see Supporting Information for details).^[^ ^33^ ^]^ The surface energy reported for Rhodamine B is much larger, that is, 55.7 mJ m^−2^, as reported elsewhere.^[^ ^34^ ^]^ During the healing of the polymer, it is more thermodynamically favorable to obtain a substrate with embedded dye than a substrate covered with the dye.

In comparison to conventional printing, painting, and stamping methods, the information inscribed on healed substrates is not directly at its surface, but at the subsurface. Logically, information registered in healed substrates are then less subjected to treatments affecting the mechanical integrity of the films such as wear and the presence of solvents. The presented process is therefore similar to the preparation of non‐temporary tattoo. Tattooing relies indeed on the insertion of dyes in the dermis layer of the skin, followed by its healing.

Data encryption in polymer was achieved by self‐healing ability of polymer and the introduction of dye in polymer. The self‐healing mechanism took place above glass transition temperature (*T*_g_) of the polymer and was affected by mobility and interdiffusion of macromolecules in polymer.^[^ ^24^, ^35^, ^36^, ^37^ ^]^ Besides, the dye in damaged surfaces can be transported through the polymer matrix due to dynamic state above *T*_g_.^[^ ^38^, ^39^ ^]^ Taking the transport of dye through the polymer, the obtainable resolution of data encryption in self‐healing polymer is limited by the diffusion of the dye. The diffusion coefficient of dyes in polymers above *T*_g_ was investigated by fluorescence correlation spectroscopy.^[^ ^39^, ^40^, ^41^, ^42^, ^43^ ^]^ The diffusion coefficient was found to belong to a wide range (≈10^−9^−10^−15^ m^2^ s^−1^) because it depends on the molecular weight of polymer, temperature of system, and polymer/dye interactions.^[^ ^39^, ^43^, ^44^, ^45^ ^]^ According to this calculation, the dye could diffuse in an area of ≈10^−4^−10^−10^ m^2^ after 24 h of healing, resulting in a lowest resolution of ≈10^−10^ m^2^. The resolution is hence limited mostly by the size of the blade or needle used for engraving the polymer.

Information storage techniques such as optical storage (DVDs or Blu‐ray discs), and magnetic storage (hard discs or magnetic tapes), have facilitated the dramatical increment of information density. Nevertheless, current information storage techniques suffered from the deterioration of storage media over time, resulting in data loss.^[^ ^46^ ^]^ Failure of storage media is typically caused by mechanical wear in hard drives, corrosion of metal layer, or oxidation of reflective layer and delamination in discs.

Therefore, sustainable methods for writing and storing information are needed. In this work, a self‐healing polymer was used as a data storage medium. The results from reversible data encryption showed that the polymeric storage medium could be reused or reprocessed several times for storing a message. Besides, the polymeric storage medium could endure polishing and extraction of solvent. Thus, our information storage technique offered the advantages in the aspects of energy‐saving and long lifetime of storage media due to self‐healing ability of material. The tattooing plastic technique could be applied to lithographic applications of nanodevices in plastic materials.^[^ ^47^, ^48^, ^49^, ^50^ ^]^ However, information density from our data storage technique is very limited, and hence required further development. We believe that the plastic tattooing technique could be applied for the preparation of electrophoretic displays and for soft electronics. For electrophoretic displays, wells and canals could be engraved and filled with dye's solution. The advantage of our material would be that the solutions could be directly engraved in one step, avoiding issues related to sealing. This type of engraving could be also applied with electronic inks so that electronic circuits could be built in one step on flexible plastics. The electronic inks would then be protected against wear and external chemical attacks because they would be embedded inside the plastics.

## Experimental Section

##### Characterization and Measurements

Gel permeation chromatography (GPC, Viscotek TDAmax, Malvern) was measured with a refractive index detector and three single‐pore GPC/size exclusion chromatography columns (6, 7, and 10 µm particle size, linear M). Polymer solutions in THF were filtered through a 0.45 mm pore size PTFE membrane filter and measured at 35 °C with a flow rate of 1 mL min^−1^. The system was calibrated with polystyrene standard. Static contact angle was measured at 25 °C. Average contact angle was obtained from at least five different locations on the films and calculated with the drop shape analysis function of the ImageJ software. Surface energy of polymer and Rhodamine B was estimated with the Owens/Wendt and Van Oss theories (see Supporting Information for details). Raman spectra of polymer film with engraved urea were recorded on a dispersive Raman microscope (Senterra II, R200‐532, Bruker) with a laser excitation wavelength of 523 nm at 20 mW with a 20× microscope magnification. The spectra were collected between 50 and 1540 cm^−1^ for 15 s of integration time at 25 °C. Dynamic mechanical analysis (DMA) was performed by EPLEXOR 150N of GABO QUALIMETER in tensile mode with a frequency of 5 Hz at temperatures from −100 to 60 °C with a heating rate of 2 °C min^−1^. Differential scanning calorimetry (DSC) was performed with a DSC (204 F1 Phoenix of NETZSCH) with a temperature range from −100 to 120 °C at a heating rate 10 °C min^−1^ under nitrogen flow. For tensile testing, the samples were prepared in the following procedure. After reaction, a diluted polymer solution was cast on a petri dish glass, and subsequently dried at 60 °C for 5 days in a vacuum oven to obtain a polymer film. Films engraved with dye were prepared by engraving the polymer films with a cutter blade, then adding Rhodamine B solution (0.25 mg mL^−1^) in the defects with a syringe, and healing at 70 °C for 24 h. The obtained engraved film with dye is shown in Figure S10, Supporting Information. The engraved films without dye were also prepared with the same procedure as for the engraved films with dye. Mechanical properties of polymer films were evaluated with an Instron 5567 Universal tester. Specimens with a width of 5.0 mm, a gauge length of 20 mm, and a thickness of 0.13 ± 0.03 mm were measured with a strain rate of 100 mm min^−1^ at 24 °C and a humidity of 55 ± 4%. The results were the average of three samples. Images of healed polymer surfaces were recorded with a dispersive Raman microscope (Senterra II, R200‐532, Bruker) with a 20× microscope magnification at 25 °C. The damage depths and the surface profiles of engraved polymer films were measured using a profilometer (Dektak XT, Bruker) with a 2 µm radius stylus at a stylus force of 0.01 mN.

##### Synthesis of Self‐Healing Polyurethane Containing Disulfide Bonds

The polymer was prepared following a slightly modified procedure.^[^ ^24^ ^]^ PTHF (polytetrahydrofuran, 20.3 g, 20.3 mmol) was first melted in a 250 mL round bottom flask at 70 °C. HDI (hexamethylene diisocyanate, 15.3 mL, 62.8 mmol) was then added into the flask and stirred to give a well‐mixed mixture, followed by addition of DBTDL (dibutyltin dilaurate, 38 µL, 0.06 mmol). After 2 h reaction, a viscous prepolymer solution was obtained and diluted with DMAc (dimethylacetamide, 80 mL) and THF (tetrahydrofuran, 20 mL). HEDS (5.1 mL, 41.7 mmol) was then charged into the solution and the reaction was allowed to proceed at 70 °C for another 16 h. *M*_n_ = 41 400 g mol^−1^ (*Ð* = 2.17).

##### Synthesis of Self‐Healing Polycarbonate‐Based Polyurethane

PCD (polycarbonate diol, 6.34 g, 6.34 mmol), HDO (1,6‐hexanediol, 0.076 g, 0.64 mmol), and HEDS (74 µL, 0.61 mmol) were melted in a 100 mL round bottom flask at 70 °C to give a well‐mixed mixture. HDI (1.20 mL, 7.49 mmol) was then added into the mixture, followed by addition of 1,4‐dioxane (15 mL). DBTDL was then added to the homogeneous mixture. The reaction was further stirred at 70 °C for 24 h. *M*_n_ = 24 900 g mol^−1^ (*Đ* = 2.08).

##### General Methods

Polymer solution after reaction was diluted with DMAc (40 mL) and THF (10 mL) to obtain a solution with low viscosity. The resulting polymer solution was cast on petri dishes or glass plates and placed in a desiccator under vacuum. Clear films were subsequently obtained after drying at 60 °C in the oven for 5 days. For the information storage, films were engraved at 25 °C with a cutter blade. For immersion tests, a solution of Rhodamine in water or Nile red in *n*‐hexane (0.025 mg mL^−1^) was deposited in the engraving. Some films were then healed at 70 °C in oven for 24 h. Then the engraved healed and non‐healed films with Rhodamine B and Nile red were placed in DI water and *n*‐hexane for 2 h, respectively. For friction tests, the polymer films were engraved with a cutter blade. Then Rhodamine B solution (0.25 mg mL^−1^) was deposited in the engraved defects. A healed film was obtained after healing at 70 °C for 24 h. A non‐healed film was prepared by deposition of Rhodamine B solution (0.25 mg mL^−1^) on the surface of a polymer film and drying at 25 °C for 2 h. The healed and non‐healed films were polished with an abrasive paper with a grit size of 1500 for 900 times. For the study of healing by Raman microscopy and profilometry, a solution (4 mL) containing PUDS polymer (43.43 g), THF (30 mL), and DMAc (120 mL) was cast on a glass slide, and subsequently dried at 60 °C for 3 days in a vacuum oven to obtain a clear polymer film with a thickness of 0.34 mm. The polymer films were engraved with defects showing a width of 28 µm, a length of 4 mm, and a depth of 2.3 µm (small defect), or a width of 130 µm, a length of 4 mm, and a depth of 82.8 µm (large defect) using a cutter blade. The engraved films were then healed at 70, 100 °C, or after dropping 0.5 µL THF on the film and treating at 70 °C. For automatic engraving process with a computer numerical control (CNC) machine, a diluted polymer solution was cast on a glass petri dish, and subsequently dried at 60 °C for 7 days in a vacuum oven to obtain a clear polymer film for further procedure. A tattoo pen clipped on a CNC drawing machine (AxiDraw V3, Evil Mad Science Laboratories, USA) was used to engrave a polymer film. The CNC machine was controlled with Axidraw extension in Inkscape software. The engraving process was operated with a tattoo pen while the CNC machine moved. For preparation of a polymer film containing two different polymers, PUDS and PCDS films were attached and bonded by dropping DMAc at the edge of the films. The joined films were heated at 60 °C for 2 days before being engraved with a cutter blade. For irreversible encryption in a composite coating, an epoxy resin was molded and used as ink reservoir. An opaque plastic layer was attached over the epoxy resin mold and an ink was injected into the gap between the opaque plastic layer and the epoxy resin mold. A self‐healing polymer topcoat was placed on the reservoir. The film was then heated for 0.5 h at 60 °C to yield a composite coating. The composite coating was engraved with a tattoo needle and then healed for 15 h to obtain an engraved message in the polymer film. The tattoo pen (Hawk rotary), tattoo needle, and tattoo ink were purchased from Solong Tattoo, China.

## Conflict of Interest

The authors declare no conflict of interest.

## Supporting information

Supporting Information

Supplemental Video 1
