# Supplementary material for: Tattooing Plastics with Reversible and Irreversible Encryption
Source: Adv Sci (Weinh). 2020 Apr 22;7(13):1903785. doi: 10.1002/advs.201903785 (PMC7341078; doi:10.1002/advs.201903785)
Supplement: Supplementary file 1 — Supporting Information [file ADVS-7-1903785-s001.pdf]

Copyright WILEY-VCH Verlag GmbH & Co. KGaA, 69469 Weinheim, Germany, 2020.

Supporting Information

Tattooing plastics with reversible and irreversible encryption

*Tiwa Yimyai, Treethip Phakkeeree, and Daniel Crespy**

Materials

Polytetrahydrofuran (PTHF, $M_n \approx 1000$ g mol^{-1}, Sigma-Aldrich) was dried at 120 °C under vacuum for 2 h before use. Polycarbonate diol (PCD, $M_n \approx 1000$ g mol^{-1}, ETERNACOLL PH100, UBE chemicals), 2-Hydroxyethyl disulfide (HEDS, ≥85.0%, Sigma-Aldrich), dibutytin dilaurate (DBTDL, 95%, Sigma-Aldrich), dicyclohexylmethane 4,4'-diisocyanate (HMDI, >90.0%, TCI Chemicals), hexamethylene diisocyanate (HDI, ≥98.0%, Sigma-Aldrich), 1,6-hexanediol (HDO, >97.0%, TCI Chemicals), Rhodamine B (C.I.45170 for microscopy, ≥90%, Merck), Nile red (TCI Chemicals), urea (>99.0%, TCI Chemicals), *n*-hexane (95%, Carlo Erba), tetrahydrofuran (THF, ≥99.9%, Carlo Erba), *N*,*N*-dimethylacetamide (DMAc, >99.0%, TCI Chemicals), 1,4-dioxane (≥99.8%, Carlo Erba), ethylene glycol (99.8%, anhydrous, Acros Organics), glycerol (≥99.5%, Carlo Erba), and *n*-octane (≥99%, Carlo Erba) were used as received. DI water was used throughout the experiments.

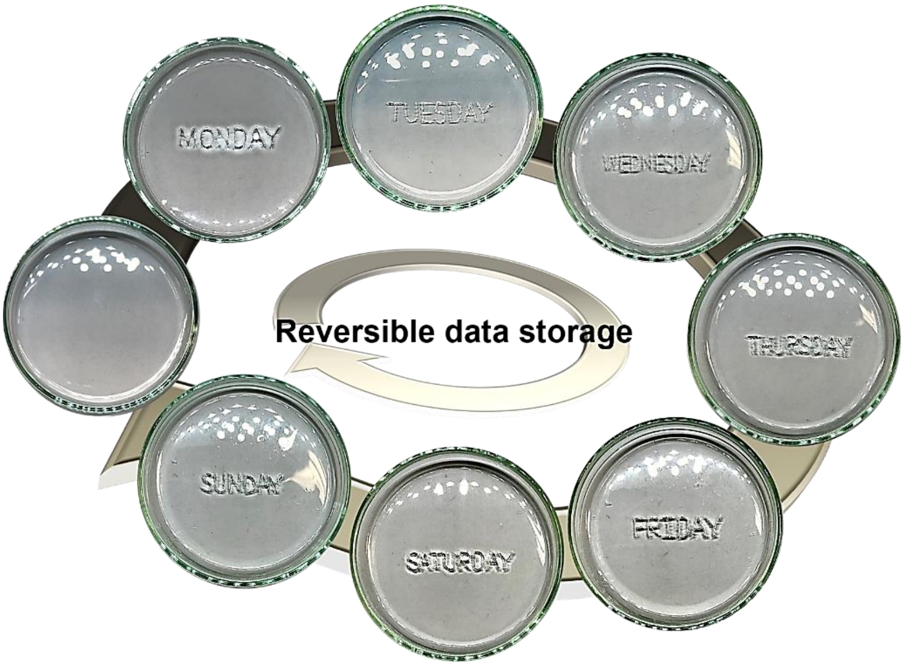

Figure S1. Photographs of reversible data storage in self-healing elastomers. The week day was engraved every day and subsequently erased by temperature increase on the same elastomeric substrate.

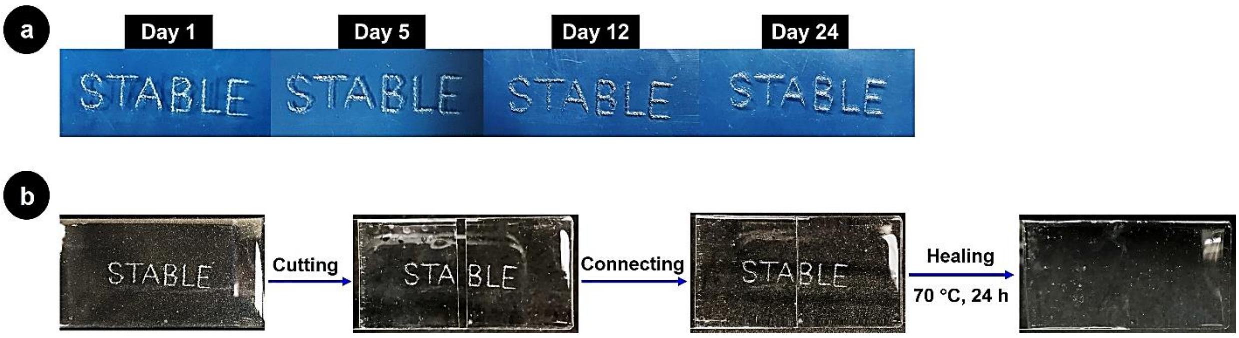

Figure S2. a) Photographs of an engraved message on a self-healing polymer film stored at 23–24 °C and a humidity of 36 ± 4 % taken at different time intervals. b) Self-healing test of the engraved film after exposure at 23–24 °C and a humidity of 36 ± 4 % for 24 days. The engraved film was cut, connected, and then healed at 70 °C for 24 h. After healing, a transparent film without scratches and engraving was obtained.

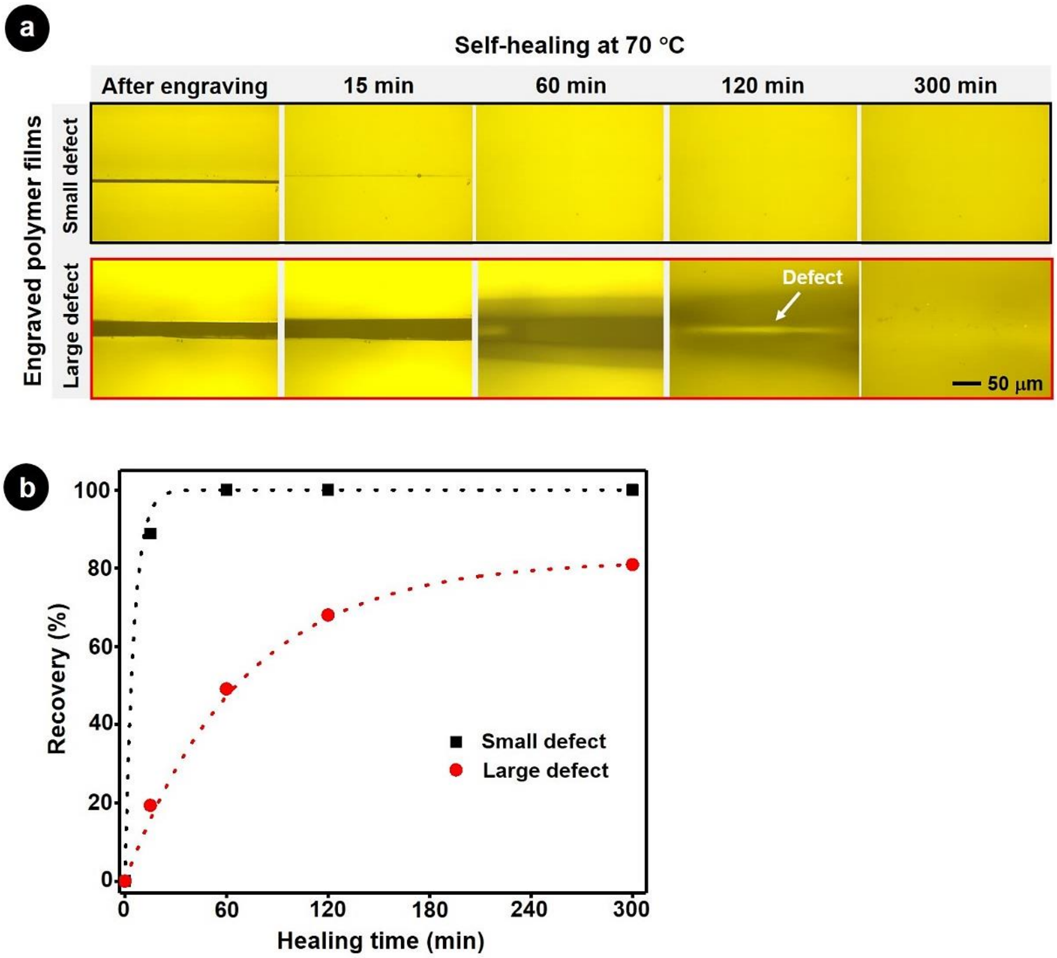

Figure S3. a) Raman microscopy time-lapse images of engraved polymer films with a small defect (28 μm × 4 mm × 2.3 μm) and a large defect (130 μm × 4 mm × 82.8 μm) healed at 70 °C. b) Temporal evolution of the recovery of damage with different types of defects on polymer films. The recovery (R) was defined as $R = (d_0\text{-}d)/d_0$, d_0 and d being the depth of the damage measured by profilometry after scratching and at various healing time, respectively.

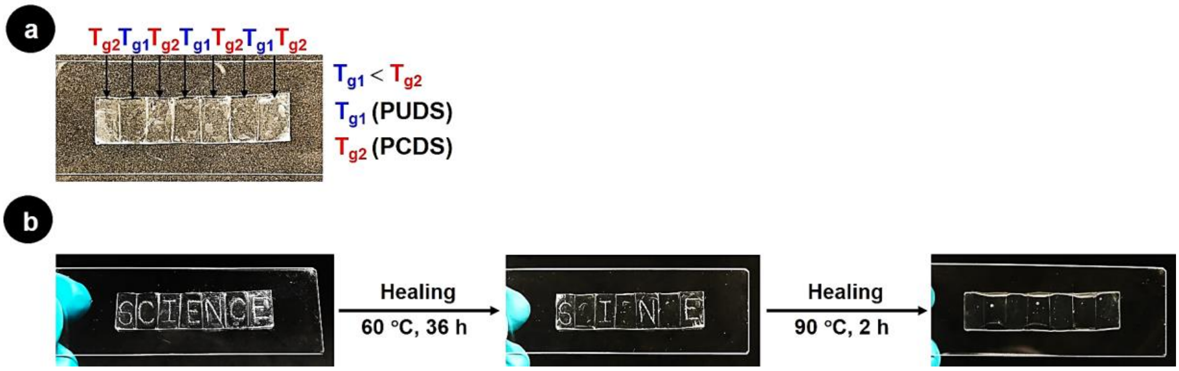

Figure S4. a) Photograph of polymer film containing different sections formed with a polyurethane (PUDS) and a polycarbonate-based polyurethane (PCDS). b) Photographs of the film with engraved word, i.e. "SCIENCE", which was transformed to "SINE", and then completely erased upon increase of temperature.

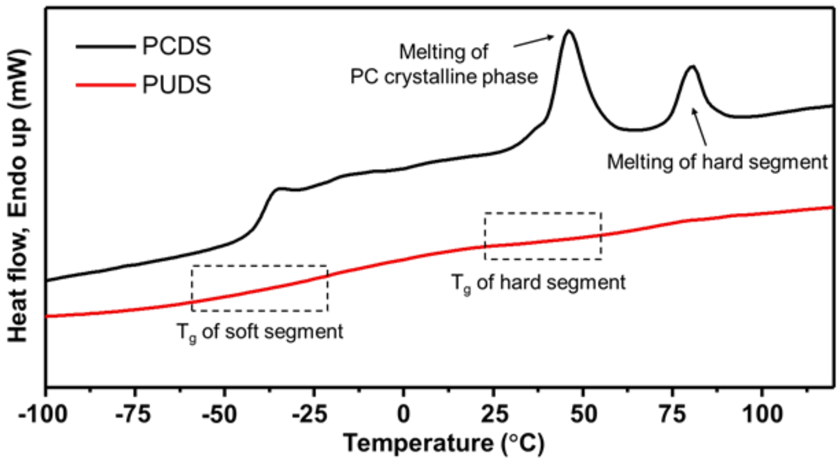

Figure S5. DSC thermogram of the polycarbonate-based polyurethane (PCDS) and the polyurethane containing disulfide bonds (PUDS) upon first heating.

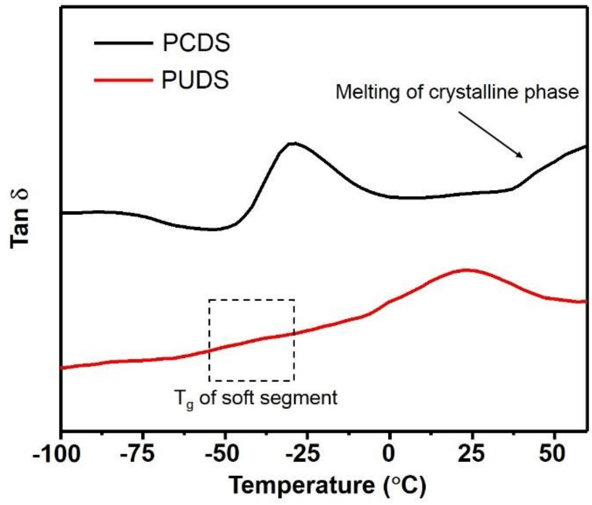

Figure S6. Tan δ of the polycarbonate-based polyurethane (PCDS) and the polyurethane containing disulfide bonds (PUDS) polymers as function of temperature determined by dynamic mechanical analysis.

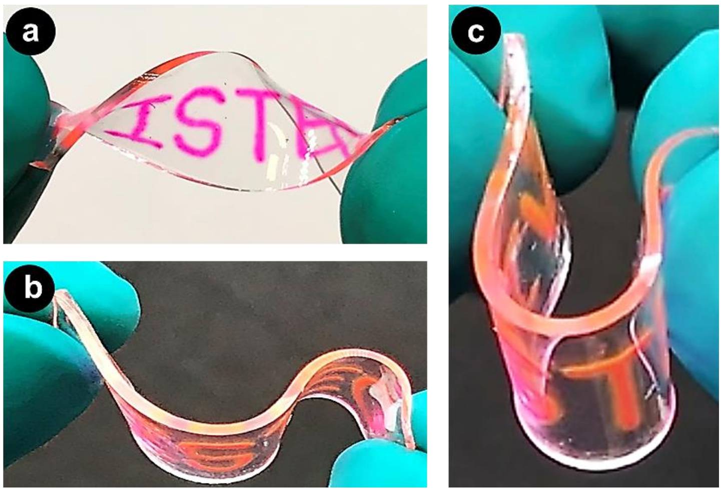

Figure S7. Photographs of bent and twisted self-healing substrates at 25 °C engraved with Rhodamine B (a-c).

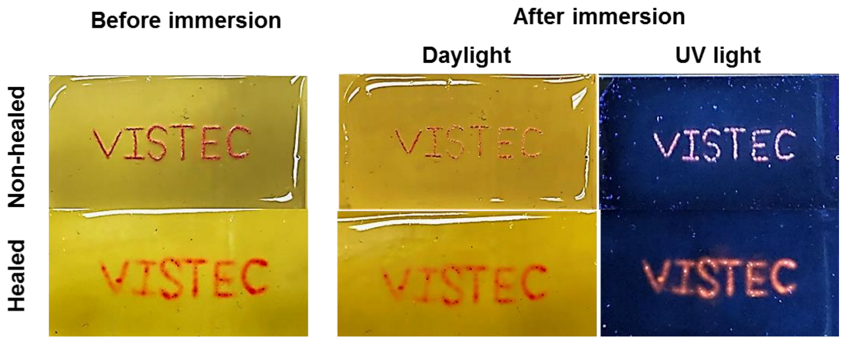

Figure S8. Photographs of substrates engraved with Nile red before and after immersion in *n*-hexane for 2 h.

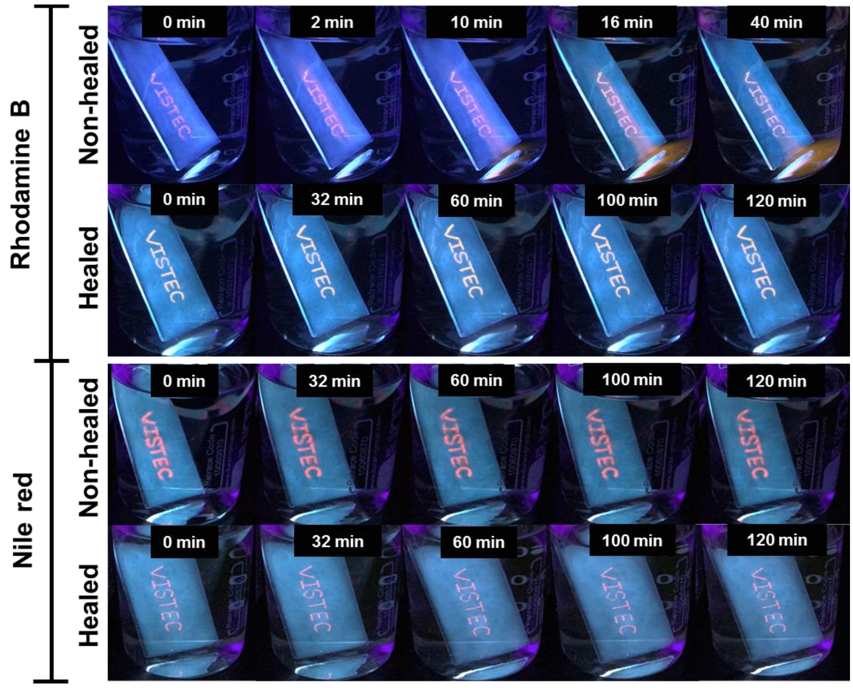

Figure S9. Time-lapse photographs of non-healed and healed films under UV light (λ = 365 nm) which were engraved with Rhodamine B and Nile red immersed in water and *n*-hexane, respectively.

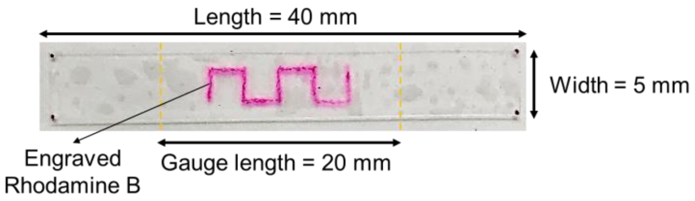

Figure S10. Polymer film engraved with Rhodamine B for tensile testing.

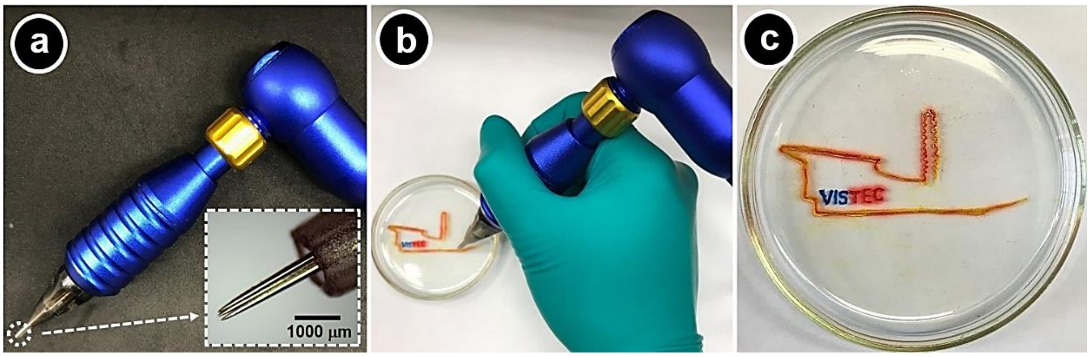

Figure S11. a) Photographs of a tattoo pen and magnified image of three-round liner (3RL) tattoo needle (inset). b) Irreversible encryption in polymer film using tattoo pen. c) Tattooed polymer film after healing.

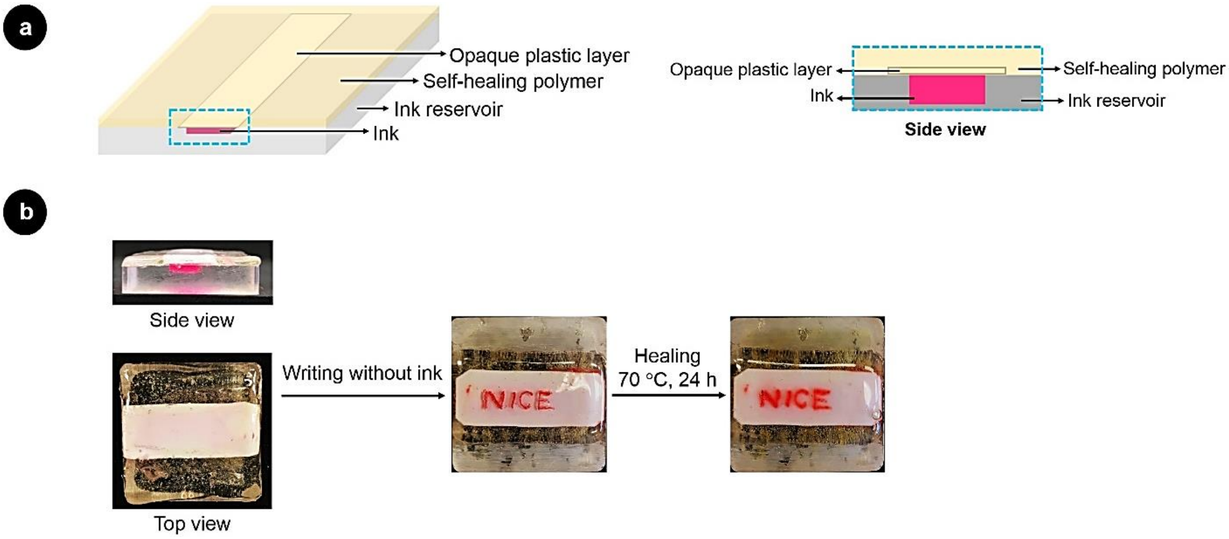

Figure S12. a) Schematic design of the composite coating. b) Preparation of an irreversible encryption in the composite structure coating.

Surface energy calculation of self-healing polymer and Rhodamine B

Estimation of surface energy of the polymer by the Owens/Wendt method

The Owens/Wendt method is typically used for determining surface energy of materials containing polar and non-polar components:[1]

$$\frac{\sigma_L (\cos\theta + 1)}{2 (\sigma_L^D)^{1/2}} = (\sigma_S^P)^{1/2} \frac{(\sigma_L^P)^{1/2}}{(\sigma_L^D)^{1/2}} + (\sigma_S^D)^{1/2} \quad (1)$$

where: θ = contact angle between liquid and solid, σ_L = overall surface tension of wetting liquid, σ_L^D = dispersive component of surface tension of wetting liquid, σ_L^P = polar component of surface tension of wetting liquid, σ_S = overall surface energy of solid, σ_S^D = dispersive component of surface energy of solid and σ_S^P = polar component of surface energy of solid. The overall surface energy of solid (σ_S) is calculated as $\sigma_S = \sigma_S^D + \sigma_S^P$.

Equation (1) can be re-written in the linear form of y = mx + c with:

$$y = \frac{\sigma_L (\cos\theta + 1)}{2 (\sigma_L^D)^{1/2}}, \; m = (\sigma_S^P)^{1/2}, \; x = \frac{(\sigma_L^P)^{1/2}}{(\sigma_L^D)^{1/2}}, \text{ and } c = (\sigma_S^D)^{1/2}.$$

Table S1. Surface tension components and contact angles on self-healing polymer at 25 °C.

Solvent	σ_L^D (mJ m^{-2})	σ_L^P (mJ m^{-2})	σ_L (mJ m^{-2})	Average contact angle on self-healing polymer (°)
DI water	21.8[2]	51.0[2]	72.8	90.3 ± 1.6
Glycerol	34.0[2]	30.0[2]	64.0	80.6 ± 1.8
Ethylene glycol	29.0[2]	19.0[2]	48.0	65.4 ± 1.4
n-Octane	21.8[3]	0.0[3]	21.8	0.0 (spreading)

The linear equation of Owens/Wendt plot was y = 1.9995x + 4.6414 (R^2 = 0.9948). The surface energy components of self-healing polymer are shown in **Table S2**.

Surface energy of the dye

Surface energy components of Rhodamine B were reported by another group to be σ_S^{LW} = 38.6 mJ m^{-2} (Lifshitz-Van der Waals apolar component), σ_S^+ = 1.42 mJ m^{-2} (acid component of surface energy of solid), and σ_S^- = 51.7 mJ m^{-2} (base component of surface energy of solid).[4] The Lifshitz-Van der Waals apolar component can be defined as dispersive component. According to the reported surface energy components, the overall surface energy can be calculated as follows:[5]

$$\sigma_S = \sigma_S^{LW} + \sigma_S^{AB} \quad (2)$$

with σ_S is the overall surface energy of solid and σ_S^{AB} is the Lewis acid-base polar component is expressed as:

$$\sigma_S^{AB} = 2\,(\sigma_S^{+}\sigma_S^{-})^{1/2} \tag{3}$$

The surface energy components of Rhodamine B are shown in Table S2.

Table S2. Surface energy components of self-healing polymer and Rhodamine B at 25 °C.

Material	σ_S^D (mJ m^{-2})	σ_S^P (mJ m^{-2})	σ_S (mJ m^{-2})
Self-healing polymer	21.5	4.0	25.5
Rhodamine B	38.6	17.1	55.7

Supporting References

[1] D. K. Owens, R. C. Wendt, *J. Appl. Polym. Sci.* **1969**, *13*, 1741.

[2] C. J. Van Oss, *Interfacial forces in aqueous media. 2nd ed.*, CRC Press, FL, USA **2006**.

[3] B. D. Summ, Y. V. Goryunov, *Physico-chemical fundamentals of wetting and spreading*, Chemistry, Moscow, **1976**.

[4] A. Ontiveros-Ortega, M. Espinosa-Jiménez, E. Chibowski, F. González-Caballero, *J. Colloid Interface Sci.* **1998**, *199*, 99.

[5] C. J. Van Oss, M. K. Chaudhury, R. J. Good, *Chem. Rev.* **1988**, *88*, 927.
